# *Streptococcus pyogenes* and EBV coinfection in severe adult meningoencephalitis: a rare diagnosis in a diabetic patient

**DOI:** 10.3389/fcimb.2025.1695084

**Published:** 2026-01-16

**Authors:** Xiaoxia Yang, Zhenzhen Li, Xiaoqing Dai, Ting Chai, Shaojun Huang, Fen Hu

**Affiliations:** Xiangyang Central Hospital, Affiliated Hospital of Hubei University of Arts and Science, Xiangyang, China

**Keywords:** EBV, intracranial infection, rare diagnosis, *Streptococcus pyogenes*, TNGS

## Abstract

**Background:**

Intracranial infection caused by *Streptococcus pyogenes* is extremely rare in adults. We report a case of *Streptococcus pyogenes* combined with Epstein-Barr virus (EBV) infection in cerebrospinal fluid (CSF), which was diagnosed by targeted next-generation sequencing (tNGS). The findings were subsequently confirmed by capillary electrophoresis (CE) and quantitative real-time PCR (qPCR), respectively. Meanwhile, digital PCR (dPCR) detection indicated the presence of *Streptococcus* species DNA in the blood. Brain magnetic resonance imaging (MRI) suggested the possibility of meningitis. The patient’s condition improved significantly following combined antibacterial and antiviral therapy.

**Case summary:**

A 67-year-old female patient with a history of diabetes mellitus for over ten years presented with fever, vomiting, and impaired consciousness. CSF analysis showed significantly elevated cell count, elevated neutrophil percentage, and increased lactate dehydrogenase (LDH) levels. The tNGS result of CSF indicated *Streptococcus pyogenes* and EBV coinfection, meanwhile, dPCR of peripheral blood suggested the presence of *Streptococcus* species. The patient was therefore diagnosed with intracranial infection, sepsis, and septic shock. The antimicrobial therapy was promptly adjusted to include combined antibacterial and antiviral treatment. Following intervention, the patient’s symptoms gradually improved. Subsequent CSF analysis and tNGS testing demonstrated normalization of cell count and LDH levels, a significant decrease in the detection level of *Streptococcus pyogenes*, and negative detection of EBV.

**Conclusion:**

Intracranial coinfection involving *Streptococcus pyogenes* and EBV is exceptionally rare in adults. Early clinical manifestations are often nonspecific, yet the disease can progress rapidly, underscoring the critical importance of prompt and accurate pathogen identification. The widespread application of tNGS has significantly enhanced early diagnostic capabilities in cases of intracranial infection.

## Introduction

Intracranial infection caused by *Streptococcus pyogenes* is highly uncommon and is associated with significant rates of morbidity and mortality (Ruth et al., 2020; Navin et al., 2022). EBV encephalitis, a serious infection of the central nervous system, is predominantly observed in children and remains relatively rare in adults (Marine et al., 2023; Wang et al., 2025). Intracranial coinfection of *Streptococcus pyogenes* and EBV is extraordinarily rare, and to the best of our knowledge, has not been previously documented in the medical literature. Herein, we present a detailed analysis and summary of a case of severe concurrent intracranial infection with *Streptococcus pyogenes* and EBV in an adult patient treated at our institution.

## Case presentation

We report a case of a 67-year-old female who presented with fever, vomiting, and impaired consciousness. According to the patient’s family, on May 20, 2025, she developed abdominal pain, diarrhea with watery stools, urinary frequency, and urgency without an obvious cause. On the morning of May 23, 2025, she experienced mild dizziness upon waking, which subsequently worsened accompanied by nausea and vomiting. Her body temperature was measured at 38.6 °C in the afternoon. She self-administered Wushicha Granules and ibuprofen capsules, but no significant reduction in fever was observed. The family immediately took her to a local hospital, where symptomatic and supportive treatment was initiated. At 2:00 PM, she developed confusion and slurred speech and was subsequently transferred to our hospital. According to the family, the patient had a more than ten-year history of diabetes mellitus and had been regularly taking metformin, acarbose, and subcutaneous insulin. No other significant personal or family history was identified.

Physical examination on admission revealed a body temperature of 38 °C, pulse rate of 100 beats/min, respiratory rate of 34 breaths/min, oxygen saturation of 100% (with nasal cannula oxygen support), and blood pressure of 110/60 mmHg. The patient was conscious; bilateral pupils were equal in size and round, with a diameter of approximately 2 mm, and the light reflex was brisk. Coarse breath sounds with scattered moist rales were audible over both lung fields. Multiple erythema were noted on the skin, accompanied by profuse sweating. The abdomen was mildly distended with slight tenderness on palpation, but no rebound tenderness was detected.

**Figure 1:**
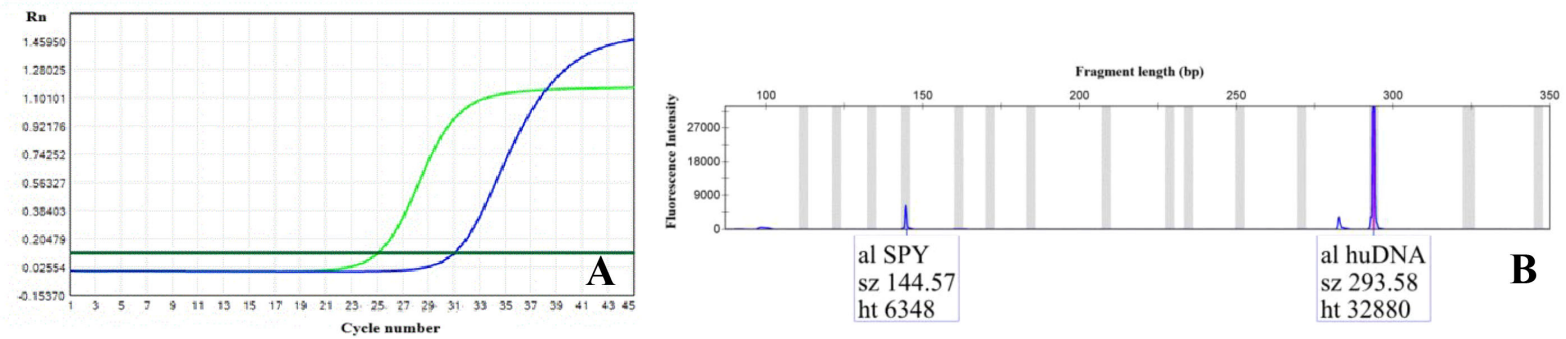
Capillary electrophoresis and qPCR results of the CSF. **(A)** The qPCR result demonstrated the presence of EBV DNA in the CSF (X-axis represents Cycle number; Y-axis represents Rn, Normalized reporter signal. The blue curve denotes EBV, and the green curve denotes the internal reference gene). **(B)** The capillary electropherogram result confirmed the presence of *Streptococcus pyogenes* nucleic acid in the CSF (X-axis represents Fragment length, bp; Y-axis represents Fluorescence intensity. The abbreviations al, sz, ht correspond to area, size and height respectively. The SPY peak represents the *Streptococcus pyogenes* gene, and the huDNA peak corresponds to the internal reference gene).

Upon admission, the patient received comprehensive management including antibiotic therapy, correction of acidosis, endotracheal intubation with mechanical ventilation, blood purification, as well as adjuvant therapies such as gastric protection and intestinal flora regulation. Plain head computed tomography (CT) scan revealed only multiple lacunar infarcts in the bilateral basal ganglia and corona radiata, with no evidence of intracranial infection or other brain injury. CSF culture was performed four times with pediatric aerobic & facultative anaerobic culture bottle (bioMérieux, BACT/ALERT PF Plus), and all results were negative. However, CSF analysis demonstrated a significantly elevated nucleated cell count with neutrophil predominance, along with markedly increased proteinorrhachia and LDH levels (**Table 1**), consistent with bacterial intracranial infection. Complete blood count showed a white blood cell (WBC) count of 16.29×10^9^/L with 95.9% neutrophils, and serum ferritin was elevated at 305.05 ng/mL, further supporting the possibility of bacterial intracranial infection. The antimicrobial regimen was adjusted to piperacillin-tazobactam (4.5 g IV q8h) and norvancomycin (800 mg IV qd). Subsequent broad-spectrum pathogen testing via tNGS (GensKey) of the CSF detected EBV at a concentration of 10^4^ copies/mL and *Streptococcus pyogenes* at 10³ copies/mL (**Table 2**), which were confirmed by qPCR (Daan gene) and CE (HEALTH BioMed), respectively (**Figure 1**). Concurrently, bloodstream infection testing via dPCR (pilotgene) indicated *Streptococcus* species infection with a load of 560.5 copies/mL (**Table 3**). And tNGS of the blood sample identified the *Streptococcus* species as *Streptococcus pyogenes*. Consequently, the patient was diagnosed with intracranial coinfection of *Streptococcus pyogenes* and EBV, accompanied by sepsis. Antiviral therapy with acyclovir (5–12.5 mg/kg IV q12–24h) was initiated. Due to an elevated serum creatinine level of 310.8 μmol/L, which suggested renal failure, piperacillin-tazobactam and norvancomycin were discontinued. The antimicrobial therapy was switched to ceftriaxone (1 g IV q12h) combined with linezolid (0.6 g IV q12h). The treatment was administered for a full week before being stopped, with no other toxic or side effects observed during the treatment period.

**Table 1:** CSF of laboratory results.

Sample no.	CSF							Blood
	Date	WBC (×10^6^/L)	Differential leukocyte count	Proteinorrhachia (g/L)	Glycorrhachia (mmol/L)	Chlorine (mmol/L)	LDH (U/L)	Glycemia (mmol/L)
1	2025/5/26	1154	Neut 83%; Lym9%; Mon8%	5.25	4.6	135.2	484	14.76
2	2025/5/28	2286	Neut74%; Lym14%; Mon12%	2.73	3.39	125.2	636	13.17
3	2025/6/16	4	/	1.43	3.36	121	24	9.9

WBC, white blood cell; Neut, Neutrophil; Lym, Lymphocyte; Mon, Monocyte;.

**Table 2:** The tNGS results of the CSF.

CSF	Date	Pathogen detected	Concentration (copies/ml)	Sequence number
1	2025/5/28	EBV	1×10^4^	663474
		*Streptococcus pyogenes*	1×10^3^	604574
2	2025/6/16	EBV	0	0
		*Streptococcus pyogenes*	<1×10^3^	24361

**Table 3:** The dPCR results of the blood.

No.	Test item	Result (copies/ml)	Reference range
1	*Stenotrophomonas maltophilia*	<25.0	0.0∼25.0
2	*Pseudomonas aeruginosa*	<25.0	0.0∼25.0
3	*Klebsiella pneumoniae*	<25.0	0.0∼25.0
4	*Acinetobacter baumannii*	<25.0	0.0∼25.0
5	*Staphylococcus aureus*	<25.0	0.0∼25.0
6	*Escherichia*	<25.0	0.0∼25.0
7	*Candida*	<25.0	0.0∼25.0
8	*Enterococcus*	<25.0	0.0∼25.0
9	*Streptococcus*	560.5	0.0∼25.0
10	*Enterobacter cloacae complex*	<25.0	0.0∼25.0
11	*Proteus mirabilis*	<25.0	0.0∼25.0
12	*Coagulase-negative staphylococci*	<25.0	0.0∼25.0
13	*Serratia marcescens*	<25.0	0.0∼25.0

**Figure 2:**
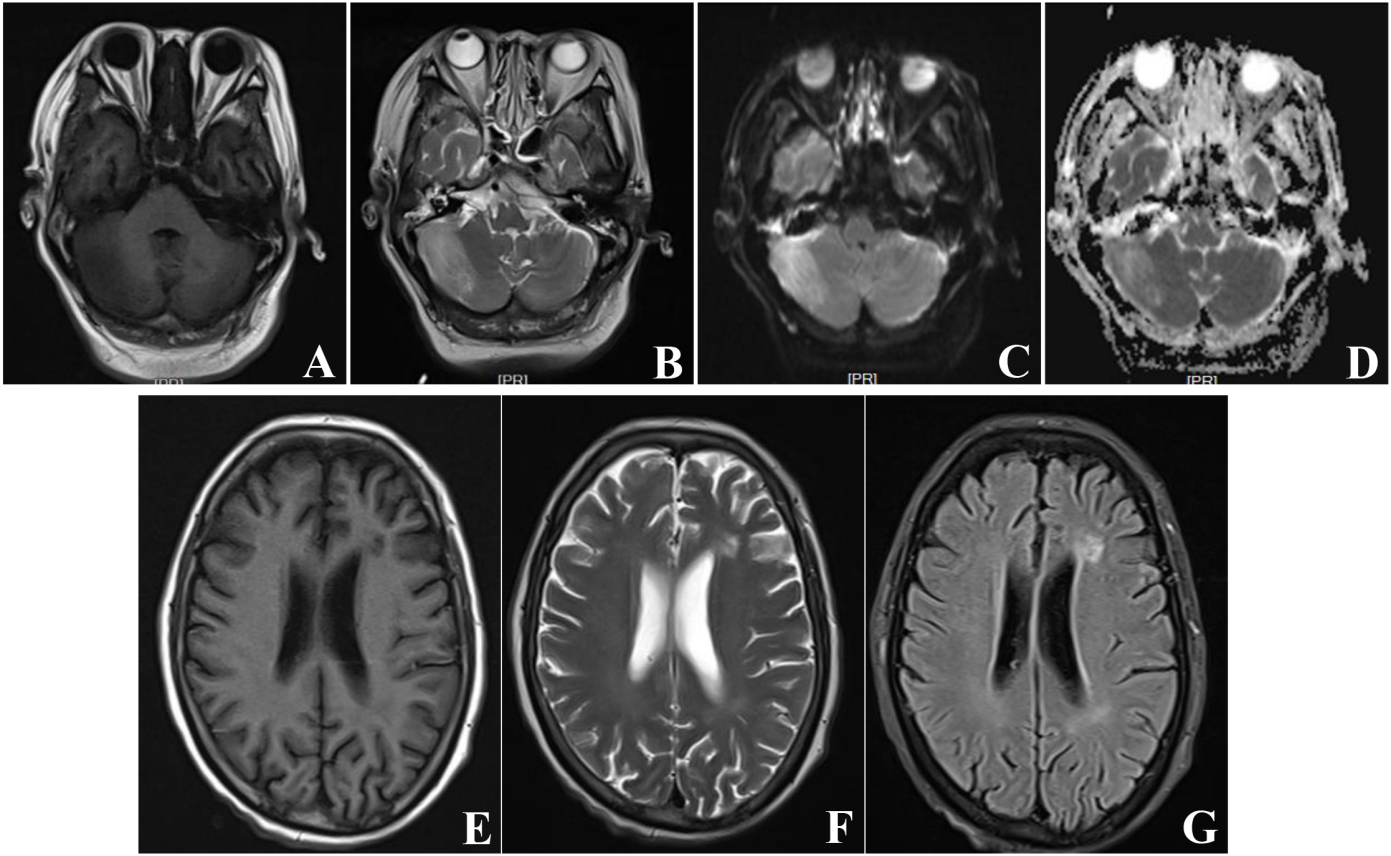
MRI images. MRI was performed after treatment. The MRI images showed the right cerebellum a patchy long T1 **(A)** and long T2 **(B)** signal shadow, with equivalent signal intensity on DWI **(C)** and a slightly longer signal on ADC **(D)**, and the left insular lobe a patchy long T1 **(E)** and slightly long T2 **(F)** signal shadow, with high signal on T2 Flair imaging **(G)**.

After one week of intensive inpatient treatment, the patient was able to open her eyes spontaneously and maintained autonomous respiration following extubation. She was largely able to cooperate with coughing and expectoration, although she remained confused and exhibited fluctuating body temperature. By two weeks of treatment, her mental status had gradually returned to normal; she was able to respond appropriately to questions, with occasional reports of dizziness and headache, and no further fever or cough. A brain magnetic resonance imaging (MRI) scan was performed to evaluate the disease status, which revealed a patchy area with prolonged T1 and T2 signals in the right cerebellum, isointense on DWI and slightly hyperintense on ADC. Contrast-enhanced imaging showed dural thickening of the right transverse sinus with homogeneous enhancement. A patchy area with slightly long T1 and T2 signals was also observed in the left insular cortex, appearing hyperintense on T2-FLAIR and demonstrating heterogeneous enhancement post-contrast (**Figure 2**). These findings, in conjunction with the clinical history, were considered suggestive of meningoencephalitis.

A follow-up lumbar puncture and CSF analysis demonstrated a marked decrease in cell count, proteinorrhachia, and LDH levels (**Table 1**). Pathogen detection via tNGS indicated a significant reduction in the concentration and read counts of *Streptococcus pyogenes*, and EBV was not detected (**Table 2**). Together, these results indicate substantial improvement of the intracranial infection.

## Discussion

The patient initially presented with abdominal pain, diarrhea with watery stools, as well as frequent and urgent urination. The condition subsequently progressed to fever and vomiting, followed by rapid neurological deterioration manifesting as confusion. CSF examination results indicated an infection with *Streptococcus pyogenes* combined with EBV. Following combination antibacterial and antiviral therapy, the patient’s mental status improved, with resolution of fever and alleviation of headache and dizziness, indicating gradual control of the disease. A follow-up lumbar puncture demonstrated normalization of CSF cell count, total protein, and LDH levels. NGS results revealed undetectable EBV DNA and a significant reduction in nucleic acid levels of *Streptococcus pyogenes*, further confirming therapeutic response.

*Streptococcus pyogenes* is a Gram-positive, host-adapted bacterial pathogen that commonly causes superficial human infections such as pharyngitis and impetigo. Although less common, it can also lead to severe invasive syndromes, including septicemia and streptococcal toxic shock syndrome (STSS) (Stephan et al., 2023). STSS is a rare, life-threatening, toxin-mediated infectious process that leads to rapid and severe shock, multiple organ failure syndrome, and death (Enora et al., 2024). In the early stage of the disease, clinical manifestations typically include fever, diarrhea, headache, and confusion, among which fever is the most common symptom, followed by non-specific gastrointestinal discomfort. Diffuse erythema is also frequently observed on the skin in the initial stages (Roman et al., 2019). *Streptococcus pyogenes* infection can further induce acute renal failure and glomerulonephritis (Barnham et al., 2002), both of which align with the clinical features observed in this case. Early recognition and prompt initiation of effective antimicrobial therapy are critical to reducing associated morbidity and mortality. Due to the non-specific nature of initial symptoms, detection of *Streptococcus pyogenes* in sterile body fluids—such as blood, CSF, or peritoneal fluid—can facilitate rapid confirmation of the diagnosis (Enora et al., 2024). In recent years, NGS has been widely adopted for rapid identification of pathogenic microorganisms in clinical samples. In the present case, tNGS of CSF successfully detected *Streptococcus pyogenes*, enabling rapid confirmation of intracranial infection and guiding timely targeted therapy.

EBV is a double-stranded DNA virus belonging to the Herpesviridae family, with tropism for B lymphocytes. It is one of the most successful human viruses, infecting over 90% of the global population and establishing lifelong persistence (Marine et al., 2023). Reactivation and proliferation of EBV can occur under conditions of immune dysregulation, potentially leading to invasion of the central nervous system (CNS) (Liu et al., 2025). Patients with EBV encephalitis most frequently present with headache and/or fever (83%), or gastrointestinal symptoms such as nausea, vomiting, and diarrhea (40%) (Marine et al., 2023). Neurological disturbances, particularly confusion and impaired consciousness, are observed in approximately 35% of cases (Marine et al., 2023). The clinical features of EBV encephalitis are non-specific and often indistinguishable from those of other infectious encephalitides. Thus, nucleic acid detection in CSF remains a cornerstone for definitive diagnosis (Whitley and Gnann, 2002). In the present case, intracranial EBV infection was confirmed by tNGS of the CSF, followed by validation using qPCR.

An association between diabetes and increased susceptibility to infection has long been recognized. Individuals with diabetes face a 1.5- to 4-fold higher risk of infection due to several host factors, including hyperglycemia-induced immune dysfunction, vascular insufficiency, peripheral and autonomic neuropathy, and increased colonization of skin and mucous membranes by pathogens (Richard et al., 2024). The patient in this case had a long-standing history of diabetes, which likely contributed to a heightened vulnerability to pathogenic infection. Moreover, STSS occurs predominantly in elderly patients aged 50–69 and is frequently associated with underlying comorbidities such as diabetes, malignancy, and cardiac conditions (Lepoutre et al., 2011; Plainvert et al., 2012). Therefore, we hypothesize that immunocompromised status—particularly in the context of diabetes—may have facilitated intracranial co-infection with both *Streptococcus pyogenes* and EBV, thereby triggering the subsequent cascade of clinical symptoms observed.

The widespread clinical adoption of tNGS has significantly advanced the diagnostic landscape for intracranial infections. However, due to the rarity of *Streptococcus pyogenes* as a causative agent in such infections, some tNGS assays targeting the CNS may lack sufficient coverage or optimized workflows for reliable detection of this pathogen. This case underscores that intracranial infection with *Streptococcus pyogenes* can indeed occur in immunocompromised adults or in the context of co-infections with other pathogens such as EBV, highlighting the need for heightened clinical vigilance and improved pathogen coverage strategies to prevent the risk of missed diagnosis.

## Conclusion

Intracranial coinfection involving *Streptococcus pyogenes* and EBV is exceptionally rare in clinical practice. The initial clinical manifestations are often non-specific, yet the disease follows an aggressive course with significantly elevated mortality. In such cases, prompt and accurate clinical assessment, appropriate diagnostic testing, early detection, and timely initiation of targeted treatment are critical to patient survival and the reduction of morbidity and mortality. The tNGS, which includes coverage for *Streptococcus pyogenes*, exhibits substantial value in the early etiological diagnosis of CNS infections by overcoming the limitations of conventional methods, enabling precise pathogen identification that facilitates timely targeted therapy.

## Data Availability

The original contributions presented in the study are included in the article/supplementary material. Further inquiries can be directed to the corresponding author.
