# Correction: *Streptococcus pyogenes* and EBV coinfection in severe adult meningoencephalitis: a rare diagnosis in a diabetic patient

**DOI:** 10.3389/fcimb.2026.1796968

**Published:** 2026-02-23

**Authors:** Xiaoxia Yang, Zhenzhen Li, Xiaoqing Dai, Ting Chai, Shaojun Huang, Fen Hu

**Affiliations:** Xiangyang Central Hospital, Affiliated Hospital of Hubei University of Arts and Science, Xiangyang, China

**Keywords:** EBV, intracranial infection, rare diagnosis, *Streptococcus pyogenes*, TNGS

Author “Xiaoxia Yang” was erroneously assigned as corresponding author. The correct corresponding author is “Shaojun Huang and Fen Hu”.

The original version of this article has been updated.

